# Power-law rheology controls aftershock triggering and decay

**DOI:** 10.1038/srep36668

**Published:** 2016-11-07

**Authors:** Xiaoming Zhang, Robert Shcherbakov

**Affiliations:** 1 Department of Earth Sciences, University of Western Ontario, London, Ontario, N6A 5B7, Canada; 2 Department of Physics and Astronomy, University of Western Ontario, London, Ontario, N6A 3K7, Canada

## Abstract

The occurrence of aftershocks is a signature of physical systems exhibiting relaxation phenomena. They are observed in various natural or experimental systems and usually obey several non-trivial empirical laws. Here we consider a cellular automaton realization of a nonlinear viscoelastic slider-block model in order to infer the physical mechanisms of triggering responsible for the occurrence of aftershocks. We show that nonlinear viscoelasticity plays a critical role in the occurrence of aftershocks. The model reproduces several empirical laws describing the statistics of aftershocks. In case of earthquakes, the proposed model suggests that the power-law rheology of the fault gauge, underlying lower crust, and upper mantle controls the decay rate of aftershocks. This is verified by analysing several prominent aftershock sequences for which the rheological properties of the underlying crust and upper mantle were established.

Aftershocks are ubiquitous in nature. They are the manifestation of relaxation phenomena observed in various physical systems. In one prominent example, they typically occur after large earthquakes^1^,^2^,^3^,^4^. They also occur in other natural or experimental systems, for example, in solar flares^5^,^6^, in fracture experiments on porous materials^7^ and acoustic emissions^8^, after stock market crashes^9^,^10^, in the volatility of stock prices returns^11^, in internet traffic variability^12^ and e-mail spamming^13^, to mention a few. The observed aftershock sequences usually obey several well-defined, non-trivial empirical laws in magnitude, temporal, and spatial domains^4^,^14^,^15^,^16^. In many cases their characteristics follow scale-invariant distributions^16^,^17^,^18^. The occurrence of aftershocks displays a prominent temporal behavior due to time-dependent mechanisms of stress and/or energy transfer.

In the studies of seismicity, aftershock sequences are observed after moderate to large main shocks. Empirical observations reveal that aftershocks obey power-law scaling with respect to their energies (seismic moments) which in magnitude domain can be modelled by the Gutenberg-Richter law^19^. The decay rate of aftershocks above a certain magnitude is typically inversely proportional to the time since the main shock and is approximated by the Omori-Utsu law^1^,^2^:


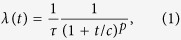


where *τ* and *c* are characteristic time scale parameters and the exponent *p* defines the power-law decay rate. This is one of the oldest and robust statistical features of aftershocks^1^,^20^ and signifies a time delay in the stress field relaxation after a main shock. Understanding the physical mechanisms responsible for the occurrence of aftershocks remains one of the fundamental problems of earthquake physics.

In this work, we consider a simple slider-block model to mimic the behavior of a seismogenic fault. We use a quasi-static approximation to map the model into a cellular automaton. In the model, we introduce a nonlinear viscoelastic coupling mechanism to capture the essential characteristics of crustal rheology and stress interaction between the blocks and the medium. For this purpose, we employ nonlinear Kelvin-Voigt elements consisting of an elastic spring and a dashpot assembled in parallel to introduce viscoelastic coupling between the blocks and the driving plate. By using the quasi-static approximation, we derive a functional form for the stress transfer mechanism in the model. We perform computer simulations of the cellular automaton realization of the slider-block model on a square grid to study its dynamics and analyze the statistics of avalanches. We show that the nonlinear viscoelasticity plays a critical role in the generation of aftershocks. It explains the functional form of the Omori-Utsu law and gives physical interpretation of its parameters.

Deviations from elastic behavior play an important role in the upper layer of the Earth^21^,^22^,^23^. This becomes evident when one considers seismogenic processes over longer characteristic time scales compared to the typical times of the propagation of seismic waves. For short time-scales classical seismology is dominated by linearly elastic treatment of the rock medium. However, the occurrence of aftershocks and other transient regimes of seismic activity are observed over much longer time-scales and signify a strong deviation from linear elasticity. There are compelling evidences that the lower continental crust and upper mantle are governed by various solid state creep mechanisms^24^,^25^,^26^,^27^. Among those mechanisms a power-law viscous flow was suggested to explain the postseismic surface deformation after large earthquakes^28^,^29^,^30^,^31^. The power-law rheology assumes that the strain rate 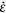 depends on stress *σ* raised to a power *n*, 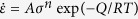, where *A* is a pre-factor, *Q* is activation energy, *R* is the universal gas constant, and *T* is temperature. The rheology of the fault zone can also exhibit deviations from elastic behavior with elements of creep and/or poroelastic deformation^32^,^33^,^34^,^35^,^36^. These observations constitute the basis for our consideration of the nonlinear viscoelastic mechanism responsible for the triggering of aftershocks. The proposed slider-block model captures these several essential characteristics of the rheological behaviour of a seismogenic fault embedded into solid material, which exhibits elements of power-law rheology. In the model, the coupling of the slider-blocks to the driving plate through the nonlinear viscoelastic Kelvin-Voigt elements represents the viscoelastic influence of the lower crust and upper mantle. The nonlinear viscoelastic coupling between the slider-blocks accounts for the complex rheology of a fault zone over longer time scales.

The original one dimensional slider-block model was introduced to simulate the occurrence of earthquakes along a fault^16^,^37^. The model was successful in reproducing the frequency-magnitude statistics of slip events^38^,^39^. Later, it has been mapped into a cellular automaton, which is known as the Olami-Feder-Christensen (OFC) model^40^,^41^. The OFC model displays several important characteristics found in earthquake phenomenology^16^,^42^,^43^,^44^,^45^. The equivalence of the slider-block model driven by the end block, which is known as a train model, and the Edwards-Wilkinson model of interface propagation was established^46^. The basic mechanisms needed for the existence of the realistic frequency-magnitude statistics and aftershock decay rates were considered in the model with the effects of contact ageing between elastic plates^47^. The importance of linear viscoelastic coupling in the slider-block type models was also recognized^48^,^49^,^50^,^51^,^52^. Viscous dumping introduces characteristic time and length scales into the problem and allows to consider a continuum limit of the slider-block type models^45^,^51^,^53^. Introduction of viscoelastic effects, in order to study the relaxation phenomenon, has been recently performed in the context of the viscoelastic interface moving in a depinning potential^54^,^55^. The decay rates of aftershocks consistent with the Omori-Utsu law, Equation (1), was observed in one particular variant of the model^55^, although, with the exponent *p* higher than empirically observed ones. The nonlinear viscoelasticity was also considered in a modified version of the Carlson-Langer model^56^. One dimensional chain of blocks was considered and numerical simulations revealed that nonlinear viscoelasticity plays a crucial role in recovering the functional form of the Omori-Utsu law with realistic values of the parameter *p*.

The existence and identification of aftershocks in the previously studied slider-block or OFC type models remains an open question. The observations that the aftershocks decay according to the Omori-Utsu law, Equation (1), have been reported in several works^42^,^43^,^44^,^45^,^57^. However, the studied models do not always possess a well defined physical mechanism for aftershock generation involving time dependent non-elastic effects. The observed time decaying rate of aftershocks can also be an artifact of the ways the rates were constructed and analyzed. The realistic decay rates of aftershocks were observed in a model where an *ad hoc* power-law stress transfer mechanism was specified^58^.

## The nonlinear viscoelastic slider-block model

**Figure 1:**
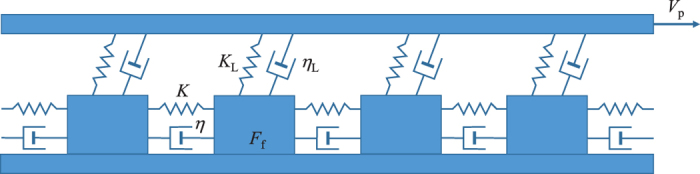
One-dimensional sketch of the model. A two-dimensional cellular automaton version of the model is used in the analysis. The slider-blocks are interconnected by nonlinear Kelvin-Voigt viscoelastic elements with parameters *K* and *η*. The blocks are placed on a surface with a simple static friction *F*_f_. The blocks are also connected to the top plate, which is driven at constant velocity *V*_p_, with similar Kelvin-Voigt viscoelastic elements with the corresponding parameters *K*_L_ and *η*_L_.

We consider a two-dimensional array of *N* × *N* slider-blocks of equal mass *m* interconnected by nonlinear Kelvin-Voigt viscoelastic elements (Fig. 1). These elements model the power-law rheology of the medium and consist of an elastic spring characterized by a spring constant *K* assembled in parallel with a nonlinear viscous dashpot characterized by a parameter *η* and a power-law exponent *n*. The blocks are placed on a surface with simple static friction. They are also connected to the top loading plate with similar viscoelastic elements characterized by the parameters *K*_L_ and *η*_L_. To perform computer simulations of the model, we use a quasi-static approximation to map the model into a cellular automaton. This model differs from the classical slider-blocks by the addition of the nonlinear viscous dashpots for the interaction between blocks and connection to the driving plate. Therefore, the nonlinear viscoelastic elements model the power-law rheology of the medium. The slow driving of the model is specified through the movement of the top driving plate with velocity *V*_p_ along a fixed direction.

The slider-blocks arranged as a two-dimensional square array of size *N* × *N* obey the equations of motion


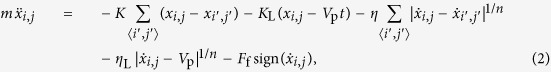


where *x*_*i*,*j*_ is a displacement of the (*i*, *j*) block from its equilibrium position, where *i*, *j* = 1,..., *N*. We assume that the movements of blocks occur only in one direction parallel to *V*_p_. *K*_L_ and *η*_L_ define the parameters of the viscoelastic elements coupling the blocks to the driving plate. The summations are performed over the nearest-neighbour blocks 〈*i*′, *j*′〉 connected to the given block (*i*, *j*). We assume a constant frictional force *F*_f_ acting on each block from the lower plate and its direction is always opposite to the block velocity 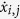.

The model is driven by the slowly moving top plate with the velocity *V*_p_. When the total force acting on a particular block from both the nearest-neighbour blocks and the driving plate exceeds the frictional force the block slips. The moving block may cause slippage of some of the nearest-neighbour blocks. Those blocks in turn can initiate more slipping events creating an *avalanche* in the system. The nonlinear viscoelastic term ensures that the slipped blocks will continue transferring the remaining stress to their nearest-neighbours as time evolves. This can cause further slipping events due to this delayed action in time.

To analyze the model, we consider a quasi-static approximation (see Methods), where we assume that the inertia term in Equation (2) is negligible. We compute the change in the value of the force acting on a given block before and after the block slips during an avalanche. This allows us to estimate the amount of stress the block transfers to its nearest-neighbour sites. Due to the presence of the nonlinear viscoelastic terms the process of stress transfer consists of the instantaneous phase and the time dependent phase. This can be summarized for the power exponent *n* ≠ 1 as follows


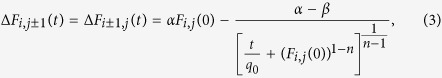


where Δ*F*_*i*,*j*±1_(*t*) is the cumulative change of stress on two nearest neighbour sites (*i*, *j* + 1) and (*i*, *j* − 1) due to the slip of the block (*i*, *j*). *F*_*i*,*j*_(0) ≥ *F*_f_ = 1.0 defines the stress acting on the block (*i*, *j*) before the slip, which is set to zero when the slip starts, *F*_*i*,*j*_(0+) = 0. *t* is a local time elapsed since the slip event and


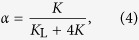



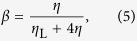



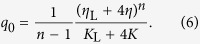


The non-dimensional parameter *α* is related to the ratio of the elastic properties of both types of springs. Similarly, the parameter *β* defines the contribution to the viscous behavior of the model due to a fault and surrounding medium. The characteristic time *q*_0_ defines the time scale of viscous relaxation. At the local time *t* = 0 the stress on the site (*i*, *j*) is reduced instantaneously to zero. At the same time the nearest-neighbour sites receive the amount of stress Δ*F*_*i*,*j*±1_(0) = Δ*F*_*i*±1,*j*_(0) = *βF*_*i*,*j*_(0). However, as the local time *t* is advanced the slipped site (*i*, *j*) will continue transferring the stress to its nearest neighbours according to Equation (3). This constitutes the fundamental mechanism of stress transfer in the model.

In the case of the linear viscoelastic elements with *n* = 1 one can find an exponential functional form for the cumulative stress transfer


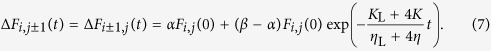


The values of the dimensionless model parameters *α* and *β* are given in the ranges 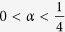 and 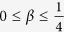. In addition, the physical considerations dictate that the cumulative stress transfer function, Equation (3), has to be an increasing function of time with a limit 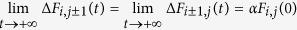. This assumes that *α* > *β* and *n* > 1. The rate of stress transfer can be obtained from Equations (3) or (7) by taking the derivative *d*[Δ*F*_*i*,*j*±1_(*t*)]/*dt*. This results in a power-law decaying rate with exponent 1 + 1/(*n* − 1) for *n* > 1.

With above considerations, we can implement the model as a cellular automaton. We simulate the model on a two-dimensional square lattice of size *N* × *N*. Each site of the lattice (*i*, *j*) is assigned a continuous stress variable *F*_*i*,*j*_ ≥ 0, which specifies the total force acting on this site (Fig. S1 in Supplementary Information). The simulation of the model starts from the uniform random distribution of forces *F*_*i*,*j*_ in the range [0, 1]. The model is driven uniformly with a constant rate until one of the sites reaches a critical threshold value *F*_*i*,*j*_ ≥ *F*_f_ = 1. The site becomes unstable and begins transferring stress to its 4 nearest-neighbours according to Equation (3). At the same time its stress is reduced to a random value very close to zero. This is done to introduce stochasticity into the model. The initial stress transfer at the local time *t* = 0 can trigger slips of its nearest-neighbours initiating an instantaneous avalanche. In addition, the slippage of unstable sites initiates the time dependent process of stress relaxation in the model as each slipped site starts its own time-dependent process of relaxation given by Equation (3) (Fig. S2 in Supplementary Information).

In the model, we adopt a limit of zero velocity driving (*V*_p_ → 0) assuming that the global loading is resumed when all relaxations cease. At that step we reset the local time *t* to zero and initiate the relaxation process due to the avalanche triggered by the global loading. During the relaxation phase between global loadings more instantaneous avalanches can be triggered. These triggered events are the result of time dependent stress transfer due to the previously slipped blocks. This constitutes fundamental nature of the model. Therefore, in the model we distinguish two types of events, i.e. the ones that are initiated by the global loading and the events initiated by the triggering mechanism due to nonlinear viscoelastic relaxation of the medium. The global loading events can be identified with background events considered in seismology. A global loading event can initiate a triggered sequence of avalanches but it is not necessarily the largest event in the sequence. It is possible that the largest event in the sequence can occur later in time. We define the largest event in a sequence as a *main shock*. Therefore, we can identify the events which precede the main shock as *foreshocks* and the events that occur after the main shock as *aftershocks* in accordance with terminology adopted in statistical seismology.

## Simulation Results

We performed numerical simulations of a cellular automaton realization of the above introduced model in order to analyze its behavior for different parameter values. The simulations were performed on a 256 × 256 square lattice. In all cases we allowed the model to pass its transient regime before the statistics were collected and analyzed. This was done by analyzing the model state. Similar to the OFC model^59^ our model displays the tendency to organize into a steady state with well defined patches of similar stress. When these patches start to dominate the system we define the end of the transient regime and start collecting statistics. In the model it is possible to identify three time scales, i.e., related to the slow global loading, to the relaxation phase associated with triggered avalanches, where the stress transfer is specified by Equations (3) or (7), and the time scale during which the avalanches propagate, which we assume to be instantaneous.

The algorithm to simulate the model also involves a nontrivial task of properly reproducing the relaxation phases between global loading events. During each such phase the time and the stress values of every slipped site have to be stored in order to allow the time dependent redistribution of stresses to their nearest-neighbours be properly accounted for as the local time evolves. In the algorithm the aftershock times are computed exactly by solving a nonlinear algebraic equation to find time needed to bring at least one site to its critical value during the relaxation of stresses from all already slipped sites. For long sequence containing large avalanches this can involve a significant number of slipped sites and usually slows down the simulations.

**Figure 2:**
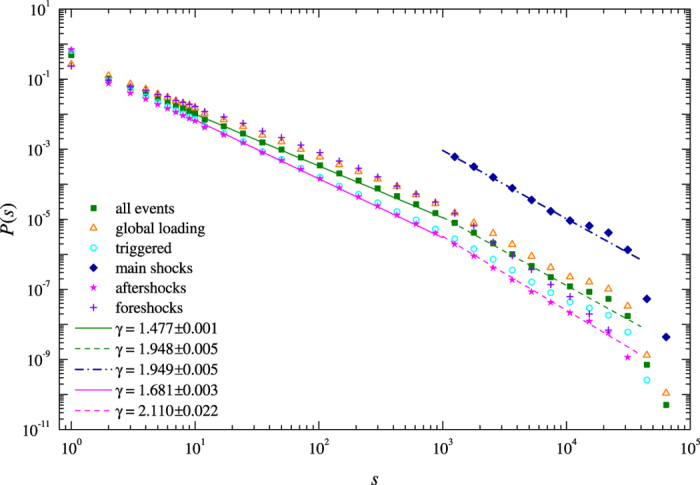
Frequency-size statistics of avalanches for the model on 256 × 256 lattice with the following model parameters: *α* = 0.24, *β* = 0.23, *q*_0_ = 10.0 and 1/*n* = 0.1. The symbols correspond to: all avalanches (squares), global loading events (open triangles), triggered events (open circles), main shocks (diamonds), aftershocks (stars), and foreshocks (crosses). The straight lines are maximum likelihood fits of the power-law function, Equation (8), and the exponent *γ* is reported within 95% confidence intervals.

One of the important characteristics of the model is the frequency size distribution of its avalanches. We define the size of an avalanche *s* as the total number of slipped sites, which form a connected cluster. We analyzed the distributions of all events and also for avalanches triggered during the relaxation process. This is shown in Fig. 2 for the following model parameters: *α* = 0.24, *β* = 0.23, *q*_0_ = 10.0, and 1/*n* = 0.1. In Fig. 2 we give the distribution of all avalanches, global loading events and triggered avalanches. For a different values of the parameters see Fig. S3 of Supplementary Information. We also distinguish between foreshocks, main shocks, and aftershocks. This is done by analyzing each triggered sequence and selecting the largest event in the sequence which has the size larger than 1000. All events after that event form aftershocks and all events before it define foreshocks. The distributions show well defined multi-scaling regimes for moderate (10 ≤ *s* < 1000) and large avalanches (*s* ≥ 1000) and each regime can be approximated by a power-law


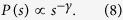


The corresponding values of the exponent *γ* are given in the legend. We attribute the appearance of multi-scaling regimes to the competing effect of elastic and viscous terms given through the parameters *α* and *β*. Each instantaneous event is characterized by the local dissipation given by the parameter *β*, whereas the cumulative effect of total dissipation during the relaxation phase is controlled by *α*. The finite-size effects are also present for large avalanches and can influence the values of the exponent *γ*. This is consistent with the behaviour of the OFC model where the degree of conservation influences the frequency-size statistics of avalanches.

**Figure 3:**
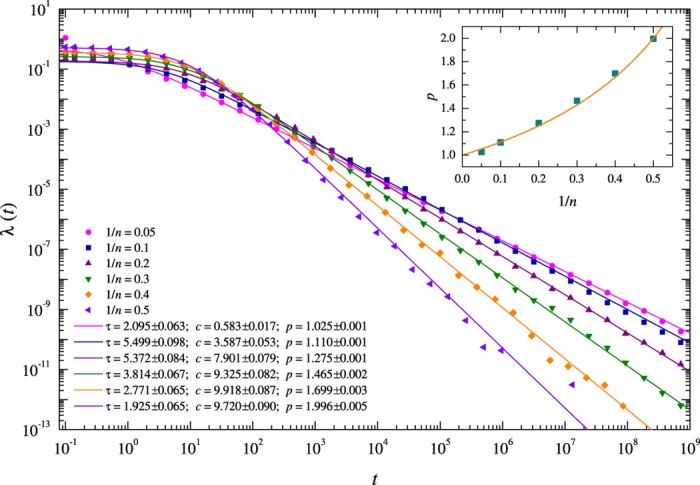
Aftershock decay rates for the model on a 256 × 256 lattice with *α* = 0.24, *β* = 0.23, *q*_0_ = 10.0, and varying 1/*n* parameters. Aftershocks larger than *s* ≥ 10 are used. The maximum likelihood fits of Equation (1) to the rates are given as solid curves and the resulting fitting parameter values are reported in the legend within 95% confidence intervals. The inset gives the dependence of the exponent *p* in Equation (1) on the model parameter 1/*n*, which defines the degree of viscoelastic nonlinearity. The solid curve corresponds to the theoretical dependence of the power exponent of the stress decay rate on *n*, which is given in Equation (9).

Another crucial measure is the decay rate of the number of aftershocks per unit time after a main shock. In case of earthquakes this rate can be approximated by the Omori-Utsu law, Equation (1), and plays an important role in studies of seismicity. Our model simulations indicate that the same functional form can be used to approximate the decay rate of the model aftershocks (Fig. 3). The rates are computed by stacking all the aftershock sequences triggered by main shocks of sizes larger than 1000 and the aftershocks which are larger than 10. In addition, the simulations show the dependence of the decay exponent *p* on the power exponent *n* of the nonlinear viscoelasticity adopted in the model and given in the inset of Fig. 3. This can be approximated well by


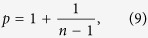


which defines the exponent of the stress decay obtained from the cumulative transfer rate, Equation (3). This also indicates that *p* > 1 for bare aftershock sequences. This is consistent with the assumption that the cumulative number of aftershocks has to be finite. A similar analysis was performed for foreshocks and their rate follows a similar functional form given by Equation (1). The obtained dependence of the parameter *p* on the degree of non-linearity *n* is consistent with the estimates derived from the numerical simulations of the one-dimensional modified version of the Carlson-Langer model^56^.

The analysis of the model for the other values of the parameters indicates that decreasing values of *α* and *β*, which is related to the degree of energy dissipation during each slippage of blocks, results in the same power-law decaying form for the aftershock/foreshock rates. In addition, there is an increase in the number of aftershocks in the early times after the main shocks. On the other hand, the difference *α* − *β* influences the productivity of aftershock sequences. The full analysis of the model for varying parameters and the finite-size effects will be reported elsewhere.

### Application to natural aftershock sequences

**Figure 4:**
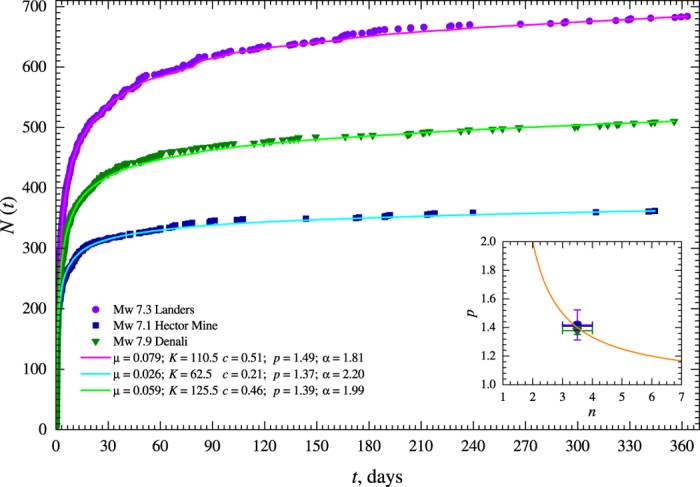
Cumulative number of aftershocks for the 1992 Mw 7.3 Landers, the 1999 Mw 7.1 Hector Mine, California, and the 2002 Mw 7.9 Denali, Alaska, main shocks are plotted as solid symbols. Aftershocks larger than *m* ≥ 3.0 and during 1 year after the main shocks are used. The maximum likelihood fits of the ETAS model, Equation (15), to the rates are given as solid curves and the resulting fitting parameter values are reported in the legend. The inset plots the mean *p* values for each sequence averaged over several magnitude cutoffs (2.8, 2.9, ..., 3.5), which were used to fit the rates, versus the power-law exponent *n* obtained from the postseismic surface deformation^29^,^60^. The vertical error bars correspond to one standard deviation of the variability of the *p* values estimated for the several lower magnitude cutoffs. The estimated parameters are given in Tables S1–S3 in Supplementary Information. The solid curve is the plot of Equation (9).

As an illustration of the applicability of Equation (9), we consider several well-known aftershock sequences for which the postseismic surface relaxation was analyzed and the rheological properties of the underlying lower crust and upper mantle were modelled^29^,^60^. For the 1992 Mw 7.3 Landers, the 1999 Mw 7.1 Hector Mine, California, and the 2002 Mw 7.9 Denali, Alaska, main shocks the power-law exponent *n* = 3.5 ± 0.5 was suggested to model the rheology of the lower crust and upper mantle^29^,^60^. For these aftershock sequences we estimated the *p*-value by fitting the Epidemic Type Aftershock Sequence (ETAS) model^61^ (see Methods). This is shown in Fig. 4. The ETAS model was used to minimize the effects of higher order aftershocks and background seismicity. The fits of the Omori-Utsu law, Equation (1), are given in Fig. S7 of Supplementary Information. Using Equation (9) one can obtain the corresponding power-law exponent *n* in the range [3.0, 3.7] which is very similar to the values obtained from the postseismic relaxation. The spatial distributions of the aftershocks, which occurred in close proximity to the main rupture planes, for each sequence are given in Figs S4–S6 of Supplementary Information.

However, we stress that the obtained relationship, Equation (9), between the power-law exponent *n* and the *p*-value of the Omori-Utsu law is valid for aftershock sequences not affected by the nonzero tectonic loading and for aftershocks occurring on cloze proximity to the main shock rupture planes. Real aftershock sequences have much more complex temporal and spatial structure. As a result, their rates can differ from ones predicted using Equation (9). However, the proposed model suggests that the power-law rheology of the fault gauge and underlying lower crust and upper mantle controls the decay rate of aftershocks.

## Discussion

In this work, we have proposed a mechanical model to illustrate the mechanisms of triggering and time delay in the occurrence of aftershocks. The model is successful in reproducing several key features of aftershocks observed in natural seismicity. The frequency-magnitude statistics displays power-law scaling for wide range of parameter values. The derived physical mechanism of stress relaxation reproduces properly the decaying rate of aftershocks in accordance with the Omori-Utsu law. We have also obtained that the parameter *p* of the Omori-Utsu law is related to the power-law exponent *n* as given in Equation (9). The results indicate that for the first time we provide a clear mechanism for the aftershock generation that follow a power-law decay rate and give a physical interpretation of its functional form. The obtained results highlight the importance of nonlinear viscoelastic effects operating in various systems exhibiting relaxation phenomena and can stimulate relevant empirical observations and experiments in order to detect and quantify such effects.

## Methods

### Quasi-static approximation

To map the slider-block model into a cellular automaton, we consider a quasi-static approximation of the dynamics of the model. In this approximation, we analyze the change in force acting on a single block after it starts slipping assuming that all other blocks did not move. Using the equations of motion, Equation (2), the change of force on the nearest-neighbour block (*i* − 1, *j*) due to the slipped block (*i*, *j*) can be written as follows:


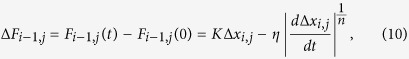


where Δ*x*_*i*,*j*_ = *x*_*i*,*j*_(*t*) − *x*_*i*,*j*_(0) is the slip of the block (*i*, *j*). The local time *t* defines the evolution of the block after it slips. This is a timescale which is much shorter than the timescale associated with the global loading given by the upper plate velocity *V*_p_ and much longer than the occurrence of instantaneous avalanches. We also assume that displacements and velocities of all the neighbour blocks are zero and consider the limit of zero velocity driving *V*_p_ → 0.

Similarly, one can consider the change in force acting on the block (*i*, *j*) after it slips:


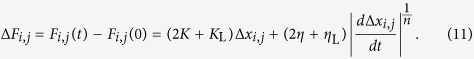


Equation (11) can be integrated to obtain the evolution of the slip:


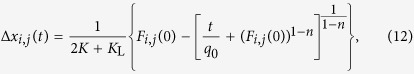


where we assume that the force *F*_*i*,*j*_ acting on the slipped block (*i*, *j*) drops instantaneously to zero. This expression for the slip evolution combined with Equation (10) can be used to obtain the equation for the change of force acting on the nearest-neighbour block (*i* − 1, *j*):


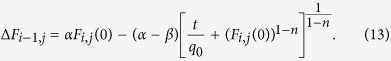


Equation (13) gives the cumulative amount of stress transferred to one of the 4 nearest neighbour blocks. Initially at *t* = 0 the nearest-neighbour block receives the amount of stress *βF*_*i*,*j*_(0). At the end, when *t* → ∞, the total amount of stress transferred is *αF*_*i*,*j*_(0) assuming that *α* > *β* and *n* > 1. The rate of stress transfer is


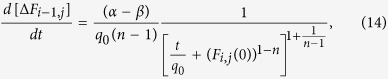


which has the same functional form as the Omori-Utsu law, Equation (1).

### The ETAS model

The decay rates of the natural aftershock sequences were modelled using the Epidemic Type Aftershock Sequence (ETAS) point process^61^. The ETAS intensity function is given by


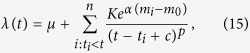


where *μ* is a background rate, *K* and *α* control the productivity of the elementary sequences, *m*_0_ is a reference magnitude, and *p* and *c* are the parameters of the Omori-Utsu law.

The parameters of the ETAS model were estimated using the maximum likelihood method by maximizing the likelihood function corresponding to the rate given by Equation (15).

## Additional Information

**How to cite this article**: Zhang, X. and Shcherbakov, R. Power-law rheology controls aftershock triggering and decay. *Sci. Rep*. **6**, 36668; doi: 10.1038/srep36668 (2016).

**Publisher’s note:** Springer Nature remains neutral with regard to jurisdictional claims in published maps and institutional affiliations.

## Supplementary Material

Supplementary Information
