# Supplementary material for: Power-law rheology controls aftershock triggering and decay
Source: Sci Rep. 2016 Nov 7;6:36668. doi: 10.1038/srep36668 (PMC5098201; doi:10.1038/srep36668)
Supplement: Supplementary Information [file srep36668-s1.pdf]

Supplementary Information for:

Power-law rheology controls aftershock triggering and decay

Xiaoming Zhang[1] and Robert Shcherbakov[1,2‡]

[1]Department of Earth Sciences, Western University,
London, Ontario, *N6A 5B7*, Canada

[2]Department of Physics and Astronomy, Western University,
London, Ontario, *N6A 3K7*, Canada

[‡]E-mail: rshcherb@uwo.ca

Model simulation

In Figure S1, we show a typical distribution of stresses in the model after it reaches a steady state. The stress field forms patches of similar values where future avalanches occur. The patch structure evolves with time. In the model each avalanche can trigger secondary avalanches. So the model has secondary and higher order aftershocks, however, they all follow the same decay rate with the same exponent. We analyze the aftershock sequences between global loadings of the driver plate, so we do not take into account the influence of the loading of the system between movements of the loading plate. This corresponds to the limit of zero velocity driving. In the model, we separate these two time scales because of computational complexity of the algorithm that simulates the cellular automaton version of the model. On the other hand, it allows us to simulate pure aftershock sequences triggered by the stress relaxation and not influenced by the background loading.

The relaxation phase of the avalanche cycle is simulated as follows. After all previous relaxations are ceased the algorithm performs the global loading of the model. It determines the largest value among the site variables, $F_{i,j}^{\max}$, in the system. Then all the sites are increased by the amount $F_{\mathrm{f}} - F_{i,j}^{\max}$, that makes the site (i, j) unstable and initiates the global loading avalanche at the local time $t = 0$. This toppled site can initiate an instantaneous avalanche of topplings of the other adjacent sites similar to the OFC model. It stores the times of failure and the actual values $F_{i,j}$ of each toppled sites which form the instantaneous avalanche (for the first avalanche all sites have the same failure time $t_0 = 0$) and the locations of their nearest-neighbour sites. When this instantaneous avalanche stops the algorithm continues to evolve the system by incrementing the local time t. Each previously toppled site continues to transfer stress to its four nearest-neighbours according to $\delta F_{i,j\pm1}(t) = \Delta F_{i,j\pm1}(t) - \Delta F_{i,j\pm1}(0)$. This stress transfer by all toppled sites can bring one of their neighbour sites above a critical stress level $F_{\mathrm{f}} = 1.0$.

To find the time t_1 needed to bring one of the neighbour sites to a critical value one needs to solve the algebraic equation for $\Delta t = t_1 - t_0$

$$\sum_{k=1}^{m} \frac{1}{\left(\frac{t-t_k+\Delta t}{q_0} + F_k^{1-n}\right)^{\frac{1}{n-1}}} = \frac{\delta F}{\beta - \alpha} + \sum_{k=1}^{m} \frac{1}{\left(\frac{t-t_k}{q_0} + F_k^{1-n}\right)^{\frac{1}{n-1}}}, \tag{S1}$$

where m is the total number of toppled sites, F_k is the value of a kth toppled site at time t_k. Here the toppled sites are arranged in a list and labeled with index k.

By knowing the time t_1 when the neighbour site $F_{i,j}^{\max}$ will fail, we increase all the neighbour sites of the toppled sites, which form the original instantaneous avalanche, by the corresponding amount of stress δF. This toppled site can initiate the first triggered instantaneous event after the global loading event. Then we continue the process of updating the model by estimating the time t_2 when the second triggered avalanche is going to happen and so on. This allows us to compute exact times of each triggered avalanche (aftershock) in the system. This significantly speeds up the simulations. Otherwise one can consider a small fixed time increment δt to advance the model between global loadings but this is inefficient. The algorithm terminates the process of stress transfer when the local time t reaches a sufficiently large value of $q_0 10^8$. By that time there are almost no triggered events in the model. After that the algorithm resumes the global loading of the model and the whole avalanche cycle starts again.

In Figure S2, we show a sequence which had a very large event, which we identify with a main shock and the sequence of smaller aftershocks. All these events happened between global loadings of the driver plate. This is a consequence of the viscoelastic relaxation adopted in the model.

We analyzed the distributions of sizes of all events and also for avalanches triggered during the relaxation process. This is shown in Fig. S3 for the following model parameters: $\alpha = 0.22$, $\beta = 0.18$, $q_0 = 10.0$, and $1/n = 0.1$. In Fig. S3 we give the distribution of all avalanches, global loading events and triggered avalanches. We also distinguish between foreshocks, main shocks, and aftershocks. This is done by analyzing each triggered se-

m_c	μ	K	c	p	α
2.8	0.100	105.26	0.547	1.553	1.512
2.9	0.091	101.53	0.513	1.505	1.627
3.0	0.079	110.52	0.513	1.495	1.808
3.1	0.060	81.05	0.471	1.472	1.799
3.2	0.051	59.30	0.365	1.407	1.832
3.3	0.024	9.86	0.053	1.259	1.372
3.4	0.023	43.77	0.311	1.355	1.999
3.5	0.023	19.10	0.128	1.301	1.743

Table S1: The estimated parameters of the ETAS model for the 1992 Mw 7.3 Landers earthquake, California, aftershock sequence using several lower magnitude cutoffs m_c to construct the rates. The sample mean value for p is 1.42 and the standard deviation 0.11.

m_c	μ	K	c	p	α
2.8	0.061	106.31	0.331	1.421	2.137
2.9	0.042	88.29	0.282	1.380	2.274
3.0	0.026	62.47	0.215	1.374	2.197
3.1	0.024	50.87	0.223	1.378	2.211
3.2	0.018	33.73	0.190	1.440	1.982
3.3	0.031	24.43	0.142	1.431	2.108
3.4	0.025	14.08	0.086	1.424	1.891
3.5	0.012	6.17	0.044	1.431	1.601

Table S2: The estimated parameters of the ETAS model for the 1999 Mw 7.1 Hector Mine earthquake, California, aftershock sequence using several lower magnitude cutoffs m_c to construct the rates. The sample mean value for p is 1.41 and the standard deviation 0.03.

quence and selecting the largest event in the sequence which has the size larger than 1000. All events after that event form aftershocks and all events before it define foreshocks. The distributions show well defined multi-scaling regimes for moderate ($10 \leq s < 1000$) and large avalanches ($s \geq 1000$) and each regime can be approximated by a power-law, Eq. (8).

m_c	μ	K	c	p	α
2.8	0.063	182.50	0.623	1.399	1.830
2.9	0.066	155.94	0.551	1.392	1.933
3.0	0.059	125.54	0.464	1.392	1.993
3.1	0.038	78.10	0.331	1.376	1.799
3.2	0.042	71.05	0.311	1.388	1.930
3.3	0.052	59.57	0.277	1.406	2.072
3.4	0.031	50.94	0.225	1.337	2.374
3.5	0.030	43.30	0.196	1.335	2.432

Table S3: The estimated parameters of the ETAS model for the 2002 Mw 7.9 Denali earthquake, Alaska, aftershock sequence using several lower magnitude cutoffs m_c to construct the rates. The sample mean value for p is 1.38 and the standard deviation 0.03.

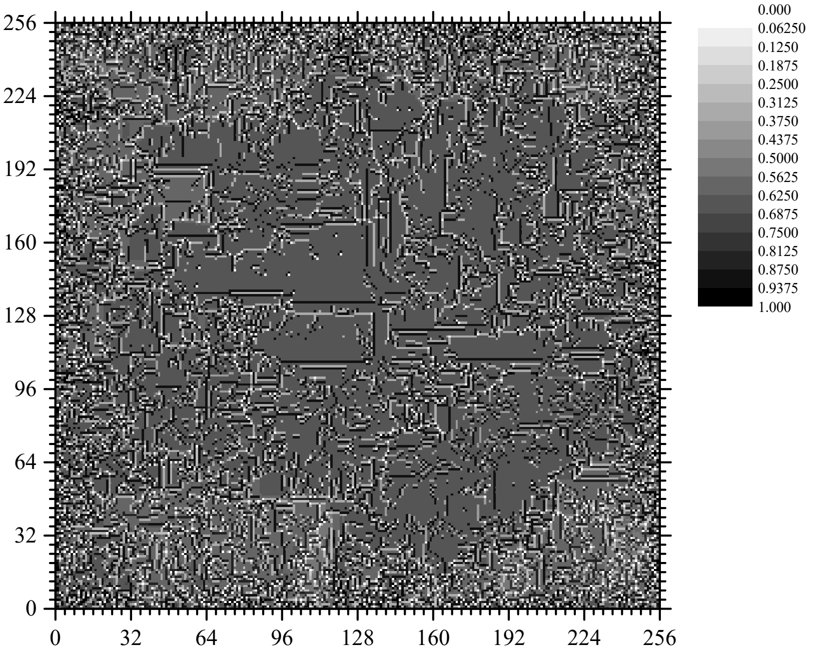

Figure S1: Typical distribution of stresses $F_{i,j}$ in the model in the steady regime. The model with parameters $\alpha = 0.24$, $\beta = 0.23$, $q_0 = 10.0$, and $1/n = 0.1$ is simulated on a 256×256 square lattice with open boundary conditions.

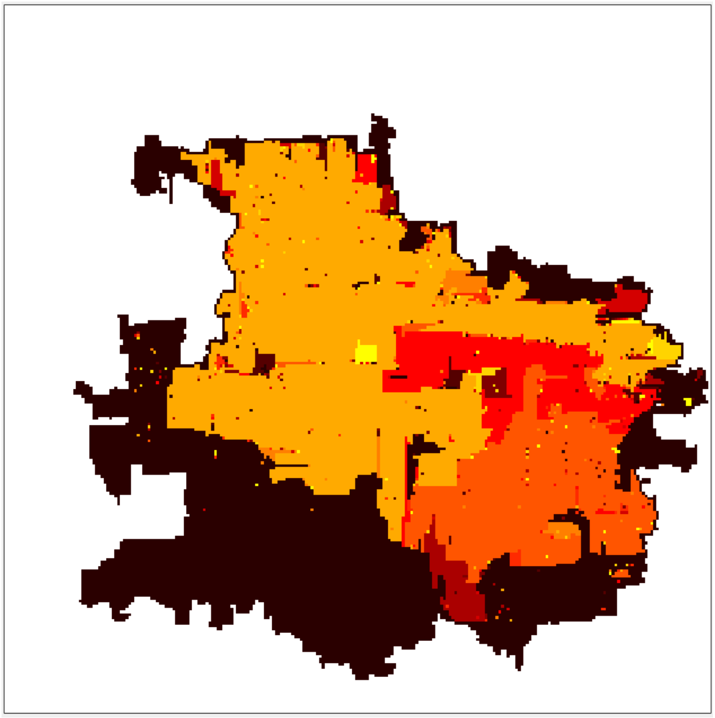

Figure S2: A sequence of aftershocks in the model containing a very large main shock. Different colors are used to illustrate all the triggered events in the model between two global loadings of the system. The model parameters are $\alpha = 0.24$, $\beta = 0.23$, $q_0 = 10.0$, and $1/n = 0.1$.

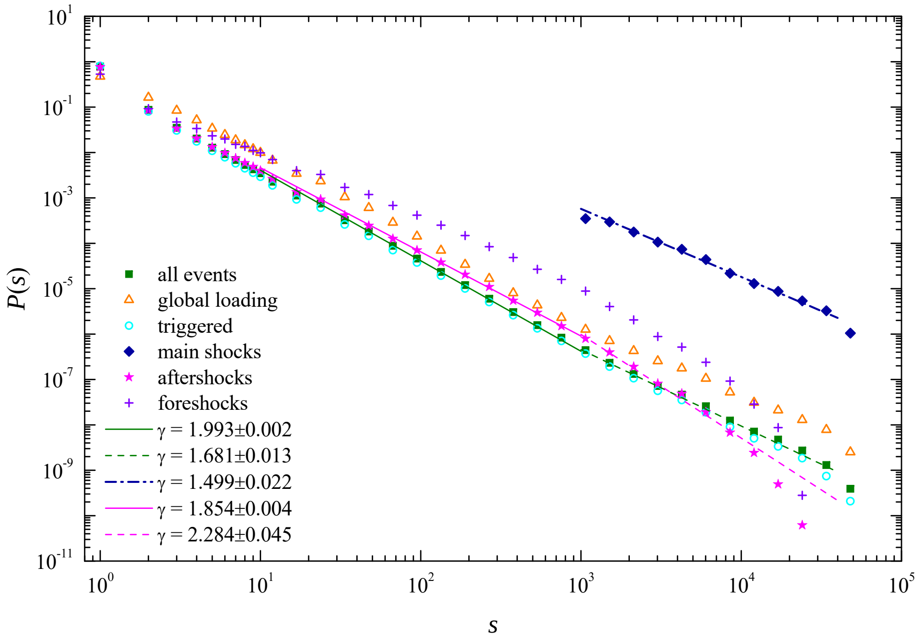

Figure S3: Frequency-size statistics of avalanches for the model on 256×256 lattice with the following model parameters: $\alpha = 0.22$, $\beta = 0.18$, $q_0 = 10.0$ and $1/n = 0.1$. The symbols correspond to: all avalanches (squares), global loading events (open triangles), triggered events (open circles), main shocks (diamonds), aftershocks (stars), and foreshocks (crosses). The straight lines are maximum likelihood fits of the power-law function, Equation (8), and the exponent γ is reported within 95% confidence intervals.

Natural aftershock sequences

In the analysis, we consider three large main shocks and their aftershocks in California and Alaska, particularly, the 1992 Mw 7.3 Landers and the 1999 Mw 7.1 Hector Mine, California, and the 2002 Mw 7.9 Denali main shocks. To extract aftershock sequences of the Landers and Hector Mine, California, main shocks, we used the relocated southern California (`http://www.data.scec.org/research-tools/alt-2011-dd-hauksson-yang-shearer.html`) earthquake catalog [1]. The aftershock sequence of the 2002 Mw 7.9 Denali main shock was extracted from the regional catalog maintained by the Alaska Earthquake Information Center (`http://www.aeic.alaska.edu/html_docs/db2catalog.html`). For all sequences we used the lower magnitude cutoff $m_c = 3.0$ and time interval of 1 year after the main shock to construct the aftershock decay rates shown in Figure 3 of the main text.

The spatial distribution of aftershocks of the three sequences considered is given in Figures S4-S6. To select aftershocks we used a spatial polygon to extract aftershocks which are in close proximity to the rupture plane of each main shock. For the Denali main shock we used the aftershocks which occurred along the main Denali fault. In most case the rupture plane of the main shocks consists of several fault segments and aftershocks are distributed both on the main shock fault and adjacent faults.

In Figure (S7) we plot the fits of the Omori-Utsu law, Equation (1), to the aftershock decay rates of the three sequences. The corresponding model parameters are also given. The obtained p values are slightly lower than ones obtained by fitting the EATS model. This is an expected result as the Omori-Utsu law approximates the decay rate of an aftershock sequences consisting of secondary and higher order aftershocks.

References

[1] Hauksson, E., Yang, W. Z. & Shearer, P. M. Waveform relocated earthquake catalog for southern California (1981 to June 2011). *Bull. Seismol. Soc. Am.* **102**, 2239–2244 (2012).

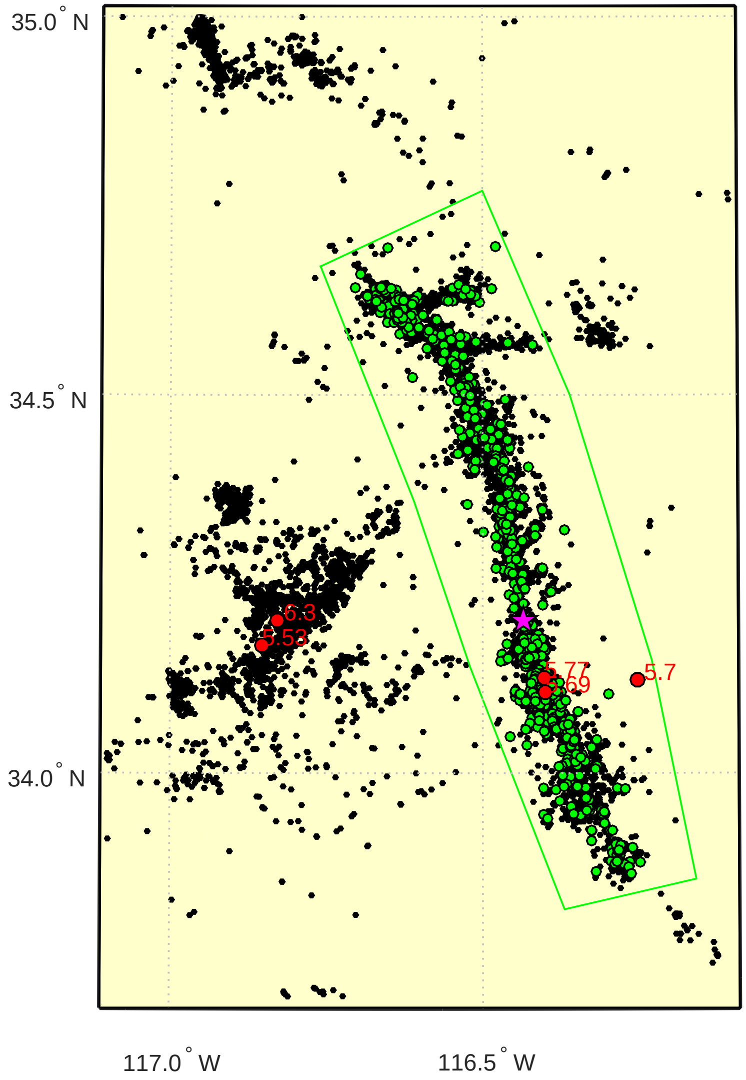

Figure S4: Spatial distribution of the aftershocks, which occurred close to the main shock rupture, of the 1992 Mw 7.3 Landers, California, earthquake. Earthquakes above magnitude $m_c = 3.0$ (green circles) and during 1 year after the main shock are shown. Earthquakes above magnitude $m_c = 2.0$ are shown as black dots. Several large aftershocks are also shown as red circles. The map was created using the Matlab Mapping toolbox, version R2016a (http://www.mathworks.com).

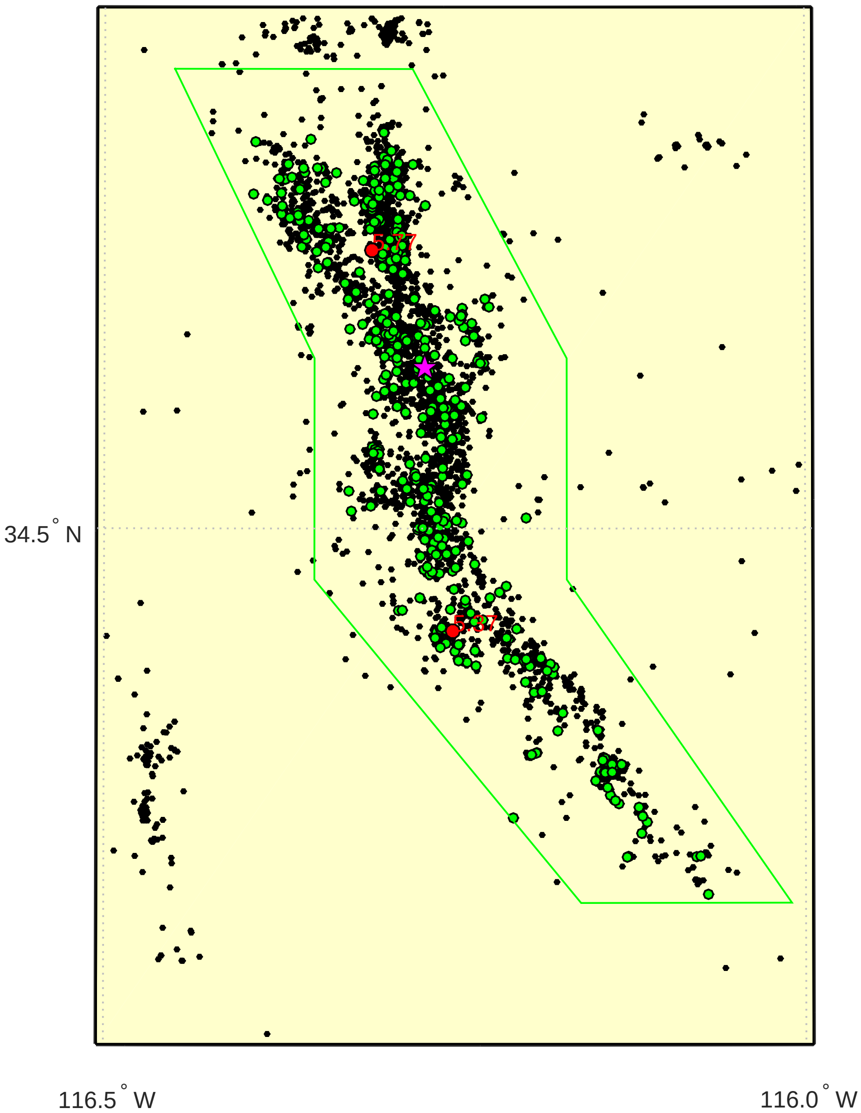

Figure S5: Spatial distribution of the aftershocks, which occurred close to the main shock rupture, of the 1999 Mw 7.1 Hector Mine, California, earthquake. Earthquakes above magnitude $m_c = 3.0$ (green circles) and during 1 year after the main shock are shown. Earthquakes above magnitude $m_c = 2.0$ are shown as black dots. Several large aftershocks are also shown as red circles. The map was created using the Matlab Mapping toolbox, version R2016a (http://www.mathworks.com).

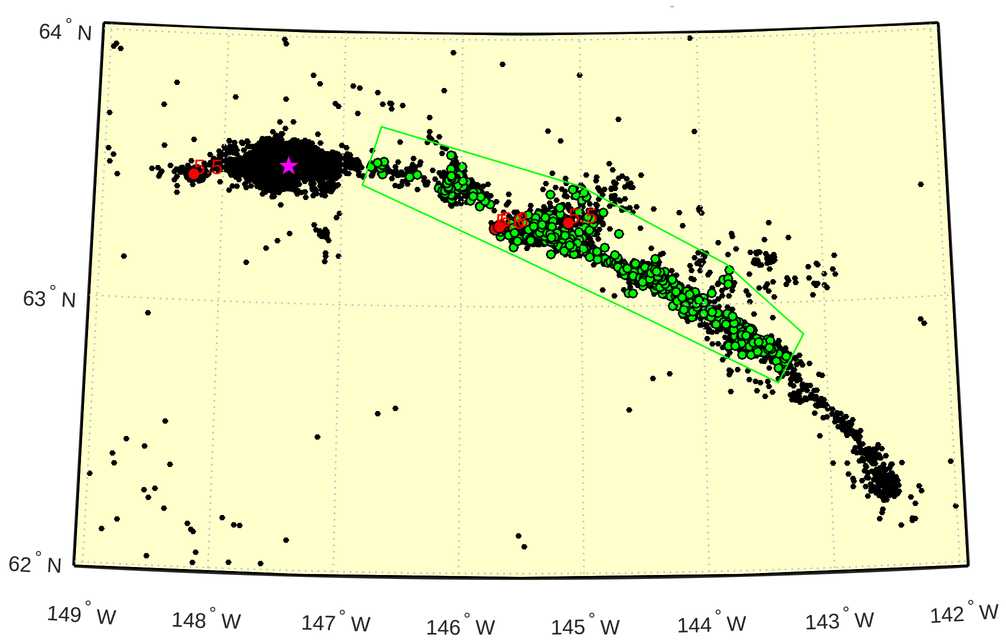

Figure S6: Spatial distribution of the aftershocks, which occurred along the rupture of the main Denali fault, of the 2002 Mw 7.9 Denali, Alaska, earthquake. Earthquakes above magnitude $m_c = 3.0$ (green circles) and during 1 year after the main shock are shown. Earthquakes above magnitude $m_c = 2.0$ are shown as black dots. Several large aftershocks are also shown as red circles. The map was created using the Matlab Mapping toolbox, version R2016a (http://www.mathworks.com).

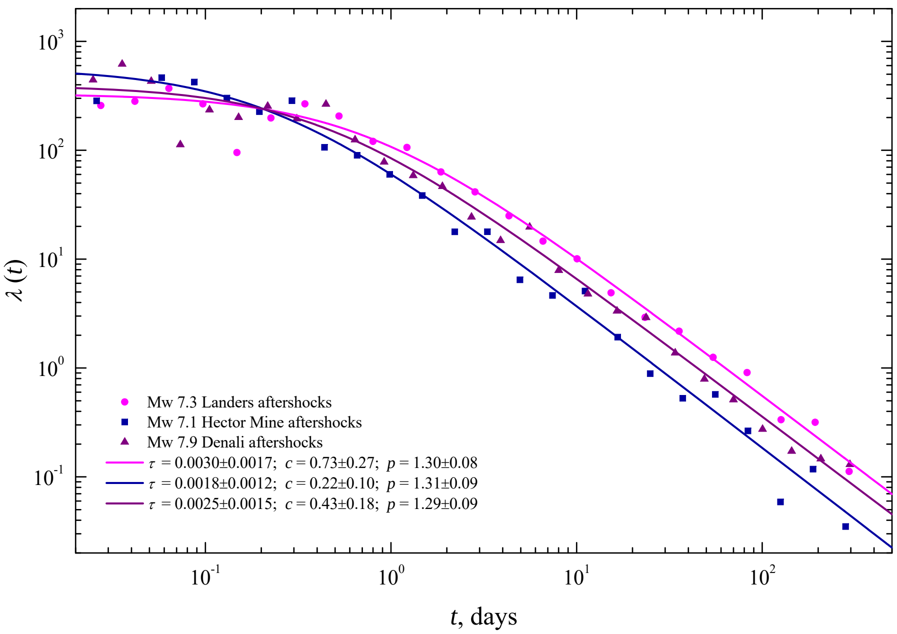

Figure S7: Aftershock decay rates for the 1992 Mw 7.3 Landers, the 1999 Mw 7.1 Hector Mine, California, and the 2002 Mw 7.9 Denali, Alaska, main shocks. Aftershocks larger than $m \geq 3.0$ are used. The maximum likelihood fits of Equation (1) to the rates are given as solid curves and the resulting fitting parameter values are reported in the legend within 95% confidence intervals.
